# Pollen-induced allergic rhinitis in the central region of Inner Mongolia, China: prevalence, risk factors, and regional characteristics

**DOI:** 10.3389/falgy.2026.1800197

**Published:** 2026-05-11

**Authors:** Ting-ting Ma, Mei-rong Yang, Zi-lu Cheng, Xiang-dong Wang, Guang-liang Shan, Yao-da Hu, Yong-fei Bai, Hui-yu Ning, Jian-ling Yang, Yan-lei Chen, Hong-tian Wang, Qing-yu Wei, Luo Zhang, Shu-lin Li, Xue-yan Wang

**Affiliations:** 1 Department of Allergy, Allergy Institute, Beijing Shijitan Hospital, Capital Medical University, Beijing, China; 2 Allergy Department, the Second People’s Hospital Ordos, Ordos, China; 3 Department of Otolaryngology Head and Neck Surgery, Beijing TongRen Hospital, Capital Medical University, Beijing, China; 4 Beijing Laboratory of Allergic Diseases, Department of Allergy, Beijing TongRen Hospital, Capital Medical University, Beijing Municipal Education Commission and Beijing Institute of Otolaryngology, Beijing, China; 5 Department of Epidemiology and Statistics, Institute of Basic Medical Sciences Chinese Academy of Medical Sciences, School of Basic Medicine Peking Union Medical College, Beijing, China; 6 State Key Laboratory of Vegetation and Environmental Change, Institute of Botany, Chinese Academy of Sciences, Beijing, China; 7 Allergy Department of Shengjing Hospital Affiliated to China Medical University, Shenyang, China

**Keywords:** allergic rhinitis, *Artemisia*, desert, grassland, skin prick test, urban

## Abstract

**Background:**

Pollen-induced allergic rhinitis (PIAR) is a major public health burden in high-pollen regions of northern China (e.g., Ordos, southern Inner Mongolia Plateau). However, regional variations in PIAR across ecological zones (urban, agropastoral, desert, and mining zones), dominant allergens, and key risk factors remain understudied due to prior small-sample or narrow-scope research.

**Objective:**

This study aimed to investigate the prevalence, major risk factors, and current treatment patterns for PIAR in Ordos.

**Methods:**

From March to July 2023, a multicenter, randomized, stratified cross-sectional survey was conducted across nine areas in Ordos. Participants were recruited to complete in-person questionnaires and undergo skin prick tests (SPTs) for 16 common allergens. Pollen was collected and counted to monitor exposure levels.

**Results:**

Among the 4,303 participants, the prevalence rates of self-reported allergic rhinitis (SRAR), physician-diagnosed allergic rhinitis (PDAR), and PIAR were 52.89% (2,276/4,303), 34.70% (1,493/4,303), and 31.51% (1,356/4,303), respectively. The prevalence rates of PIAR in urban, agropastoral, desert, and mining areas were 30.46%, 39.55%, 29.09%, and 19.72%, respectively. Among patients with PIAR, the incidence of symptom onset was highest among urban residents and lowest among mining area residents. *Poplar* pollen allergen dominated in spring, whereas in autumn, *Artemisia* pollen was predominant. Clinical symptoms were greatest in July, preceding the autumn pollen peak in September.

**Conclusion:**

PIAR is highly prevalent in northern China's grasslands, with marked zone-specific variations. *Artemisia* pollen exposure is the main sensitization driver, supporting targeted PIAR prevention/control.

## 1 Introduction

Allergic rhinitis (AR) is a major global public health concern, affecting nearly 500 million people worldwide, with its prevalence continuing to increase (1, 2). Pollen is an important allergen that triggers AR, making pollen-induced allergic rhinitis (PIAR) a global health concern. In Europe, approximately 40% of the general population is affected by pollen allergies (3, 4); in the United States, the prevalence of allergies due to ragweed pollen is 26%. Our previous survey in Inner Mongolia, China, revealed an 18.5% prevalence of PIAR (5). PIAR imposes substantial medical, social, and economic burdens on patients, families, and society.

The Ordos region, located in the southern part of the Inner Mongolia Plateau in northern China (37°24′–42°27′ N, 106°23′–111°27′ E), covers approximately 87,000 km^2,^ with an average elevation of 1,400 m (ranging from 850 to 2,149 m). The inland average annual temperature ranges from 5.3 to 8.7 °C, and the precipitation is between 190 and 400 mm. The region supports a vast array of pollen types, with the pollen concentration showing a distinct gradient from north to south (6).

Our recent research indicated that the prevalence of PIAR in Hohhot, Inner Mongolia, is as high as 32.23% (7). Because data on the prevalence of PIAR in other regions at the same latitude are lacking, this study focuses on PIAR and the dispersion characteristics of pollen in the Ordos region. However, previous research on the Ordos region has been relatively limited. For instance, the study by Deng et al. (2019) had a restricted sample size, reducing the data representativeness of the current prevalence of AR in this region (8). Additionally, Zhang et al. conducted pollen monitoring at only a few sites, introducing potential sampling biases (9). To date, no comprehensive epidemiological study has systematically examined the prevalence of PIAR among residents in this region, as well as its symptom characteristics and pollen distribution across the diverse environments of this area, including urban, agricultural and pastoral, desert, and mining zones.

In the current study, a large-scale epidemiological investigation of AR and PIAR was conducted in the Ordos region. The objectives were to estimate the prevalence rates among the local population, major clinical manifestations, sensitization patterns, and regional variations. Specifically, we aimed to assess the key risk factors for AR and analyze the symptom characteristics and concentrations of pollen across four representative areas: urban, agricultural and pastoral, desert, and mining regions. The findings provide valuable epidemiological evidence for in-depth research on AR and serve as reliable clinical references for regional prevention and control efforts.

## 2 Patients and methods

### 2.1 Study design

**Figure 1:**
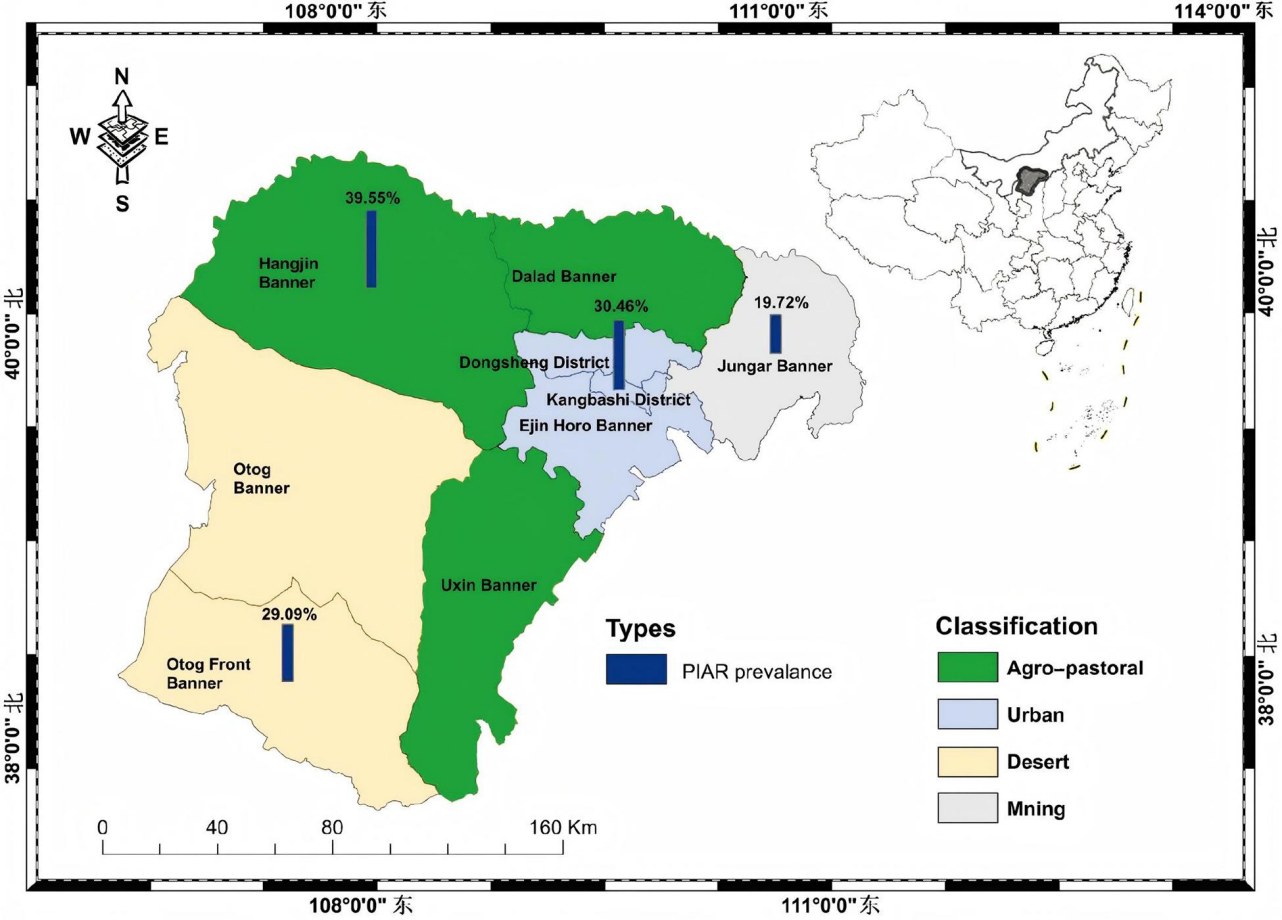
The distribution of six research areas in the ordos region, Inner Mongolia. Four distinct colors are utilized to differentiate the types of zones: agro-pastoral area (green), urban area (light blue), desert area (beige), and mining area (grey). For each zone, the bar chart illustrates the prevalence of pollen-induced allergic rhinitis (PIAR).

This study involved the populations in six areas in the Ordos region, including Dongsheng District, Kangbashi District, Hangjin Banner, Otog Front Banner, Uxin Banner, and Jungar Banner. These locations were categorized into four regional types: urban (Dongsheng District and Kangbashi District), agropastoral (Hangjin Banner and Uxin Banner), desert (Otog Front Banner), and mining (Jungar Banner) (Figure 1). The pollen counts and symptom onset patterns of patients with PIAR differed across these four regions, as described in the later sections. The Ordos region features complex topography and diverse vegetation, including typical grasslands, desertified grasslands, grassland deserts, and sandy vegetation. It has a typical temperate continental climate with distinct seasons, large diurnal temperature variations, low and unevenly distributed precipitation, and high rates of evaporation. Meteorological data were acquired from the China Meteorological Data Sharing Service Network (http://data.cma.cn/).

### 2.2 Study population

Participants were eligible for inclusion if they were aged 1–78 years and had lived in Ordos for at least 1 year. Individuals were excluded if their speech was unclear or if they were unable to cooperate with the investigation. The study consisted of two components: face‒to-face onsite questionnaire surveys and skin prick tests (SPTs). Ethical approval for this study was obtained from the ethics committee of the local hospital (Approval Number: 2021-007, Project Title: *Epidemiological Investigation and Development of Prevention and Treatment Technologies for Allergic Rhinitis in the Ordos Region*).

### 2.3 Cross-sectional study

This investigation employed a multistage stratified cluster random sampling design. The total population of the area under study was nearly 2,153,638 (urban: 693,038; rural: 1,460,600) (according to the “Erdos Region Seventh National Population Census Bulletin”, 2021).

Previous studies have reported that the estimated prevalence rates of AR among adults and children are 25.0% and 20.0%, respectively. When α=0.05 and z=1.96≈2, the minimum required sample sizes were determined to be 1,600 adult participants (800 each from urban and rural areas) and 2,000 child participants (1,000 each from urban and rural areas). There were 3,600 survey participants. The epidemiological assessment was conducted in two stages. Stage 1: Stratified sampling of administrative divisions. The six districts/banners of Ordos were first stratified into urban and rural strata based on the urban–rural population composition. Dongsheng District and Kangbashi District were designated as the urban strata. Given the diverse geomorphic characteristics of the rural stratum, it was further stratified into three geomorphic substrata: the agropastoral substratum (Hangjin Banner and Uxin Banner), the mining substratum (Jungar Banner), and the desert substratum (Otog Front Banner). Stage 2: Simple random sampling of grassroots administrative units. From each district in the urban stratum, two community residents' committees were randomly selected as sampling units. From each banner in the three rural geomorphic substrata, two village committees were randomly selected as sampling units, with all eligible residents within the selected committees invited to participate in the survey (Supplementary Figure E1).

### 2.4 Questionnaire and definition

The questionnaire was designed based on the International Study of Asthma and Allergies in Childhood, the European Community Respiratory Health Survey, and the AR and its Impact on Asthma (ARIA) guidelines (10–12). The questionnaire collected basic information from the respondents, including sociodemographic data (sex, ethnicity, age, residence, education, family income), nasal symptoms (itchy nose, runny nose, sneezing, nasal congestion), specific months during the past 12 months of the occurrence of these symptoms, related ocular symptoms (itchy or bloodshot eyes, tears), comorbid conditions (e.g., bronchial asthma, conjunctivitis, urticaria, drug or food allergies, chronic gastrointestinal diseases, hypertension, coronary heart disease, eczema, dermatitis), current treatment management, and participants' understanding of PIAR.

Two weeks prior to the study, the investigators sent letters to potential participants to introduce the study purpose and the proposed schedule. All the research subjects were reminded by the research coordinator or local health care official by phone or in person before the survey. On the survey day, participants were required to bring their IDs and sign an informed consent form. For minors younger than 18 years, informed consent was obtained from the accompanying parents or guardians. Before the study, all questionnaire surveyors and administrators for the skin prick test (SPT) received training. Each completed questionnaire and SPT report was reviewed by two people, and the results were cross-checked by the main researcher. The obtained data were independently coded by two people and entered into the database.

The diagnosis of AR followed the ARIA 2016 criteria. The subjects were asked: *Have you experienced any of the following symptoms in the past 12 months*? (i) itchy nose; (ii) sneezing; (iii) nasal discharge; and/or (iv) nasal congestion. Individuals who answered affirmatively and who had two or more of the abovementioned symptoms were diagnosed with self-reported allergic rhinitis (SRAR). For patients with SRAR, if their symptoms were triggered by local allergen(s) confirmed by SPT, a diagnosis of PDAR was made by a physician based on comprehensive clinical evaluation. The diagnosis of PIAR was based on the following criteria: ① meeting the diagnostic criteria for AR; ② a typical seasonal symptom history ≥2 years for the patient, with onset corresponding to pollen exposure; and ③ positive results from pollen allergens in SPTs (12).

### 2.5 Pollen collection and climate data

From January 1 to December 31, 2023, daily pollen count monitoring was conducted across the Ordos region. Pollen samples were collected using the gravity sedimentation method with a Durham air pollen sampler. The sampling sites were positioned 18–33 m above ground level, ensuring good ventilation across all sides and being free from major obstacles within the vicinity. Each morning at 8:00 AM, two trained technicians placed collection slides on the sampler, which were replaced every 24 h. Retrieved slides were microscopically examined under 100× and 400× magnification by two professionals to identify and quantify the types of pollen.

### 2.6 Skin prick test

SPTs were performed using standardized allergen extracts from Beijing Xinhualian Xiehe Pharmaceutical Co., Ltd. (batch number: Jingyao Zhi Zi S20130002) to detect common inhalant allergens in grassland environments. In adults, 16 specific allergens were tested: *Dermatophagoides pteronyssinus, Dermatophagoides farinae, Alternaria alternata, Felis catus, Canis familiaris, Artemisia* pollen (including *Artemisia sieversiana, Artemisia annua, and Artemisia desertorum*), *Humulus scandens, Chenopodium album, Zea mays, Helianthus annuus, Populus, Salix, Ulmus pumila, Cupressus,* and *Betula*. To reduce discomfort and potential adverse reactions in children, we appropriately reduced the number of allergens used in skin prick tests based on the results of previous local epidemiological studies and clinical experience. In children, eight specific allergens were tested: *Dermatophagoides farinae*, *Alternaria alternata*, *Artemisia sieversiana*, *Artemisia annua*, and the pollen of *Humulus scandens*, *Chenopodium album*, *Populus*, and *Cupressus*. Saline served as the negative control, and histamine (1 mg/mL) served as the positive control.

All SPTs were conducted by trained nurses on the flexor side of the unilateral forearm. After disinfection with alcohol, standardized allergen extracts were applied to prick the skin at 1.5 cm intervals. The epidermis was vertically and quickly punctured using a disposable skin test needle, after which the test solution was dropped on the skin. The participants were monitored after 15 min for symptoms such as chest tightness, dyspnea, sweating, and itching. All patients who underwent SPT had discontinued antiallergic drugs for >3 days and glucocorticoids and long-acting antiallergic drugs for >7 days. A wheal diameter of ≥3 mm was considered positive for SPT; a positive score was recorded as follows: Class 1: 3–5 mm wheal diameter; Class 2: 5–10 mm wheal diameter; Class 3: 1–2 cm wheal diameter; and Class 4: ≥2 cm wheal diameter and the presence of pseudopods.

### 2.7 Statistical analyses

Categorical variables are expressed as numbers and percentages, and continuous variables are expressed as the mean ± standard deviation (SD) or median [interquartile range (IQR)], as appropriate. Between-group comparisons of continuous variables were performed using *t* tests or the Wilcoxon rank-sum test, whereas categorical variables were compared using the chi-square test or Fisher's exact test. Multivariate logistic regression analysis was carried out to identify the risk factors associated with AR and to estimate the odds ratios (ORs). All the statistical tests were two-sided, with *p* < 0.05. All analyses were performed using SAS software version 9.4 (SAS Institute Inc., Cary, NC, USA).

## 3 Results

### 3.1 Demographic characteristics

Among the 4,800 individuals enrolled, 4,303 (89.65%; mean age, 29.81 ± 18.18 years) completed both the questionnaire and the SPT. Among them, 42.85% (1,844/4,303) were male, 57.15% (2,459/4,303) were female, 58.96% (2,537/4,303) were adults, and 41.04% (1,766/4,303) were children.

The prevalence rates of SRAR, physician-diagnosed allergic rhinitis (PDAR), and PIAR were 52.89% (2,276/4,303), 34.70% (1,493/4,303), and 31.51% (1,356/4,303), respectively. The AR prevalence did not differ significantly between males and females. Among all three types of AR, the prevalence peaked in individuals aged 18–39 years, with a gradual decrease after age 40 (*p* < 0.0001).

### 3.2 Risk factors

Mongolian participants showed significantly higher prevalence rates than Han participants did (*p* < 0.0001) (Table 1). Higher education and annual incomes were associated with increased prevalence (*p* < 0.0001 and *p* < 0.05, respectively). Civil servants and individuals working in business and services exhibited significantly higher prevalence rates than agricultural and forestry workers did (*p* < 0.0001). The prevalence of AR in individuals with a first-degree relative who had allergic diseases was greater (*p* < 0.0001). Residents of apartment buildings showed higher prevalence rates than did those living in bungalows (*p* < 0.0001) (Supplementary Table E1).

**Table 1:** Subject characteristics in SRAR, PDAR and PIAR group.

Characters	Total (*N* = 4303) n(%)	SRAR					PDAR					PIAR				
		P(95%CI)	Yes (*n* = 2276)	No (*n* = 2027)	*χ* ^2^	*P*	P(95%CI)	Yes (*n* = 1493)	No (*n* = 2810)	χ^2^	*P*	P(95%CI)	Yes (*n* = 1356)	No (*n* = 2947)	χ^2^	*P*
Age(y),(mean ± SD)	29.81 ± 18.18	52.89 (51.39, 54.39)	30.95 ± 17.44	28.53 ± 18.91			34.70 (33.27, 36.14)	28.43 ± 15.91	30.54 ± 19.24			31.51 (30.13, 32.92)	27.59 ± 15.32	30.83 ± 19.30		
Age group (y)					96.89	<0.0001				156.98	<0.0001				168.68	<0.0001
2–6y	90 (2.09)	54.44 (43.60,64.98)	49 (2.15)	41 (2.02)			42.22 (31.88,53.09)	38 (2.55)	52 (1.85)			38.89 (28.79,49.74)	35 (2.58)	55 (1.87)		
7–12y	869 (20.20)	43.27 (39.94,46.64)	376 (16.52)	493 (24.32)			30.96 (27.89,34.15)	269 (18.02)	600 (21.35)			28.88 (25.89,32.02)	251 (18.51)	618 (20.97)		
13–17y	807 (18.75)	46.96 (43.48,50.48)	379 (16.65)	428 (21.11)			31.72 (28.52,35.06)	256 (17.15)	551 (19.61)			30.24 (27.08,33.53)	244 (17.99)	563 (19.10)		
18–39y	1177 (27.35)	62.96 (60.13,65.72)	741 (32.56)	436 (21.51)			48.09 (45.20,50.99)	566 (37.91)	611 (21.74)			44.35 (41.49,47.24)	522 (38.50)	655 (22.23)		
40–59y	1,055 (24.52)	55.36 (52.30,58.38)	584 (25.66)	471 (23.24)			29.48 (26.74,32.33)	311 (20.83)	744 (26.48)			25.40 (22.80,28.14)	268 (19.76)	787 (26.71)		
≥60y	305 (7.09)	48.20 (42.47,53.96)	147 (6.46)	158 (7.79)			17.38 (13.30,22.11)	53 (3.55)	252 (8.97)			11.80 (8.41,15.96)	36 (2.65)	269 (9.13)		
Gender					0.01	0.919				3.82	0.0507				2.73	0.0987
Male	1,844 (42.85)	52.98 (50.67,55.28)	977 (42.93)	867 (42.77)			36.33 (34.13,38.58)	670 (44.88)	1,174 (41.78)			32.86 (30.72,35.06)	606 (44.69)	1,238 (42.01)		
Female	2,459 (57.15)	52.83 (50.83,54.82)	1,299 (57.07)	1,160 (57.23)			33.47 (31.60,35.37)	823 (55.12)	1,636 (58.22)			30.50 (28.68,32.36)	750 (55.31)	1,709 (57.99)		
Race					16.00	0.0003				24.68	<0.0001				30.55	<0.0001
Han	3,515 (81.69)	51.78 (50.11,53.44)	1,820 (79.96)	1,695 (83.62)			33.20 (31.64,34.79)	1,167 (78.16)	2,348 (83.56)			29.87 (28.36,31.42)	1,050 (77.43)	2,465 (83.64)		
Mongolian	690 (16.04)	59.57 (55.80,63.25)	411 (18.06)	279 (13.76)			42.90 (39.17,46.69)	296 (19.83)	394 (14.02)			40.43 (36.75,44.20)	279 (20.58)	411 (13.95)		
Other	98 (2.28)	45.92 (35.80,56.29)	45 (1.98)	53 (2.61)			30.61 (21.70,40.74)	30 (2.01)	68 (2.42)			27.55 (19.01,37.50)	27 (1.99)	71 (2.41)		
Occupation					55.27	<0.0001				54.95	<0.0001				55.91	<0.0001
Civil servant	1,211 (28.14)	61.19 (58.38,63.95)	741 (32.56)	470 (23.19)			42.69 (39.89,45.53)	517 (34.63)	694 (24.70)			39.14 (36.38,41.96)	474 (34.96)	737 (25.01)		
Business and service workers	263 (6.11)	58.17 (51.96,64.20)	153 (6.72)	110 (5.43)			31.94 (26.35,37.94)	84 (5.63)	179 (6.37)			28.52 (23.14,34.39)	75 (5.53)	188 (6.38)		
Agricultural, forestry workers	231 (5.37)	50.65 (44.01,57.27)	117 (5.14)	114 (5.62)			23.38 (18.08,29.37)	54 (3.62)	177 (6.30)			19.05 (14.19,24.71)	44 (3.24)	187 (6.35)		
Other	2,598 (60.38)	48.69 (46.75,50.63)	1,265 (55.58)	1,333 (65.76)			32.26 (30.46,34.09)	838 (56.13)	1,760 (62.63)			29.37 (27.62,31.16)	763 (56.27)	1,835 (62.27)		
Residence*						61.24	<0.0001				139.49	<0.0001			152.55	<0.0001
Dongsheng District	841 (19.54)	47.8 (44.38,51.24)	402 (17.66)	439 (21.66)			26.40 (23.45,29.52)	222 (14.87)	619 (22.03)			23.90 (21.05,26.93)	201 (14.82)	640 (21.72)		
Kangbashi District	574 (13.34)	54.18 (50.01,58.31)	311 (13.66)	263 (12.97)			44.43 (40.31,48.60)	255 (17.08)	319 (11.35)			40.07 (36.03,44.21)	230 (16.96)	344 (11.67)		
Hangjin Banner	752 (17.48)	56.12 (52.49,59.70)	422 (18.54)	330 (16.28)			36.17 (32.73,39.72)	272 (18.22)	480 (17.08)			35.11 (31.69,38.64)	264 (19.47)	488 (16.56)		
Otog Front Banner	605 (14.06)	50.58 (46.52,54.63)	306 (13.44)	299 (14.75)			30.74 (27.09,34.59)	186 (12.46)	419 (14.91)			29.09 (25.50,32.89)	176 (12.98)	429 (14.56)		
Uxin Banner	755 (17.55)	63.31 (59.76,66.76)	478 (21.00)	277 (13.67)			47.68 (44.07,51.31)	360 (24.11)	395 (14.06)			43.97 (40.40,47.60)	332 (24.48)	423 (14.35)		
Jungar Banner	776 (18.03)	46.01 (42.45,49.59)	357 (15.69)	419 (20.67)			25.52 (22.48,28.74)	198 (13.26)	578 (20.57)			19.72 (16.97,22.69)	153 (11.28)	623 (21.14)		

SRAR, self-reported allergic rhinitis; PDAR, physician-diagnosed allergic rhinitis; PIAR, pollen-induced allergic rhinitis.

Multiple regression analysis was performed to evaluate the risk factors (Table 2). Mongolian ethnicity (vs. Han ethnicity), family history of allergic conditions among first-degree relatives, cesarean delivery (vs. natural delivery), education of college or above (vs. primary school education), and residence in apartment buildings (vs. a bungalow) were all independently associated with increased risks for SRAR, PDAR, and PIAR.

**Table 2:** Risk factor analysis of SRAR、PDAR and PIAR group.

Characters	SRAR		PDAR		PIAR	
	OR(95%CI)	*P*	OR(95%CI)	*P*	OR(95%CI)	*P*
Age group		**0**.**0086**		**<0**.**0001**		**<0**.**0001**
2–6 y	Ref		Ref		Ref	
7–12 y	0.77 (0.49,1.20)		0.77 (0.49,1.22)		0.83 (0.52,1.31)	
13–17 y	0.75 (0.45,1.23)		0.62 (0.37,1.05)		0.66 (0.39,1.13)	
18–39 y	1.18 (0.71,1.96)		1.00 (0.59,1.69)		0.91 (0.53,1.56)	
40–59 y	0.95 (0.58,1.55)		0.51 (0.30,0.85)		0.44 (0.26,0.74)	
≥60 y	0.87 (0.51,1.46)		0.31 (0.17,0.55)		0.21 (0.11,0.38)	
Gender		**0**.**0699**		**0**.**0013**		**0**.**0042**
Male	Ref		Ref		Ref	
Female	0.89 (0.78,1.01)		0.80 (0.70,0.92)		0.82 (0.71,0.94)	
Race		**0**.**0023**		**0**.**0009**		**<0**.**0001**
Han	Ref		Ref		Ref	
Mongolian	1.31 (1.10,1.56)		1.38 (1.16,1.64)		1.48 (1.24,1.77)	
Other	0.73 (0.48,1.11)		0.82 (0.52,1.30)		0.84 (0.52,1.34)	
Degree of education		**0**.**0028**		**<0**.**0001**		**0**.**0001**
Elementary school	Ref		Ref		Ref	
Middle school	1.22 (0.97,1.54)		1.29 (0.99,1.69)		1.32 (0.99,1.75)	
University and above	1.63 (1.22,2.17)		1.95 (1.42,2.68)		1.95 (1.40,2.72)	
Occupation		**0**.**3312**		**0**.**0918**		**0**.**2367**
Civil servant	Ref		Ref		Ref	
Business and service workers	1.26 (0.93,1.71)		1.07 (0.78,1.48)		1.02 (0.73,1.41)	
Agricultural, forestry	1.31 (0.91,1.88)		1.56 (1.03,2.37)		1.55 (1.00,2.40)	
Other	1.15 (0.92,1.43)		1.26 (1.01,1.58)		1.14 (0.90,1.43)	
Residence		**<0**.**0001**		**0**.**0019**		
Urban	Ref		Ref			
Rural	1.44 (1.25,1.66)		1.27 (1.09,1.47)			
Annual income (CNY, ×10^4^)		**0**.**3880**		**0**.**0051**		**0**.**0277**
<5	Ref		Ref		Ref	
5∼10	1.03 (0.84,1.25)		1.04 (0.83,1.30)		1.00 (0.80,1.27)	
>10	1.12 (0.91,1.37)		1.29 (1.03,1.61)		1.21 (0.96,1.53)	
Family history		**<0**.**0001**		**<0**.**0001**		**<0**.**0001**
No	Ref		Ref		Ref	
Yes	1.81 (1.59,2.06)		1.79 (1.56,2.04)		1.84 (1.61,2.11)	
Keeping pets		**0**.**3475**		**0**.**0052**		**0**.**0036**
No	Ref		Ref		Ref	
Yes	0.92 (0.78,1.09)		0.77 (0.64,0.92)		0.75 (0.62,0.91)	
Residential environment		**0**.**0002**		**<0**.**0001**		**<0**.**0001**
Building	Ref		Ref		Ref	
Bungalow	0.72 (0.61,0.86)		0.64 (0.53,0.77)		0.67 (0.55,0.82)	
Marital status (≥18 years)						**0**.**1300**
Single					Ref	
Married					1.21 (0.95,1.54)	
WHR(Waist-to-Hip Ratio)						**0**.**5646**
Normal					Ref	
Abdominal obesity					1.06 (0.88,1.28)	
Smoking (≥18 years)		**0**.**0029**		**0**.**0066**		**0**.**0271**
Never	ref		Ref		Ref	
Current	0.91 (0.70,1.18)		0.74 (0.56,0.97)		0.70 (0.53,0.94)	
Ever	1.96 (1.26,3.05)		1.52 (0.99,2.35)		1.13 (0.71,1.79)	
Postterm Birth(<18 years)				**0**.**0809**		**0**.**1651**
No			Ref		Ref	
Yes			2.48 (0.89,6.89)		2.05 (0.74,5.63)	
Delivery mode		**0**.**0036**		**<0**.**0001**		**<0**.**0001**
Natural delivery	Ref		Ref		Ref	
Caesarean section	1.41 (1.12,1.77)		1.76 (1.39,2.23)		1.77 (1.39,2.24)	
Type of feeding		**0**.**2788**				
Breastfeeding	Ref					
Non breastfeeding	0.97 (0.65,1.45)					
Mixed type	0.85 (0.68,1.04)					
Mother smoke before pregnancy(<18years)				**0**.**0985**		
No			Ref			
Yes			0.48 (0.21,1.14)			

SRAR, self-reported allergic rhinitis; PDAR, physician-diagnosed allergic rhinitis; PIAR, pollen-induced allergic rhinitis; OR, odds ratio; CI, confidence interval; Ref, reference category.

All listed covariates were included in each regression model. For clarity, OR, 95% CI, and *p*-values are only presented for statistically significant associations (*p* < 0.05). Non-significant results are omitted from the table but were fully included in the analysis.

Note: Only *P*-values are formatted in bold for emphasis and visual clarity in the table. Bolding does not denote statistical significance; all results are presented as reported.

With respect to age-related risk factors, an inverse association was observed, with individuals aged 40 years or older being significantly less likely to develop AR and PIAR than those aged 2–6 years were (*p* < 0.0001). Women had a lower risk of developing AR and PIAR than men did (PDAR: OR = 0.80, 95% CI: 0.70–0.92, *p* = 0.0013; PIAR: OR = 0.82, 95% CI: 0.71–0.94, *p* = 0.0042). Mongolian ethnicity was associated with a greater risk of developing AR than Han Chinese ethnicity was (SRAR: OR = 1.31, 95% CI: 1.10–1.56, *p* = 0.0023; PDAR: OR = 1.38, 95% CI: 1.16–1.64, *p* = 0.0009; PIAR: OR = 1.48, 95% CI: 1.24–1.77, *p* < 0.0001).

The risk of developing AR and PIAR was significantly greater for individuals with a university degree or higher than for those with a primary school education (SRAR: OR = 1.63, 95% CI: 1.22–2.17, *p* = 0.0028; PDAR: OR = 1.95, 95% CI: 1.42–2.68, *p* < 0.0001; PIAR: OR = 1.95, 95% CI: 1.40–2.72, *p* = 0.0001). Compared with individuals residing in urban areas, those in residing rural areas had a significantly greater risk of developing AR (SRAR: OR = 1.44, 95% CI: 1.25–1.66, *p* < 0.0001; PDAR: OR = 1.27, 95% CI: 1.09–1.47, *p* = 0.0019). The risk of developing AR and PIAR was significantly lower for individuals living in bungalows than for those living in high-rise buildings (SRAR: OR = 0.72, 95% CI: 0.61–0.86, *p* = 0.0002; PDAR: OR = 0.64, 95% CI: 0.53–0.77, *p* < 0.0001; PIAR: OR = 0.67, 95% CI: 0.55–0.82, *p* < 0.0001). Compared with never-smokers, ever-smokers had a significantly greater risk of developing SRAR (OR = 1.96, 95% CI: 1.26–3.05, *p* = 0.0029), whereas current smokers demonstrated a reduced risk of developing PDAR/PIAR (PDAR: OR = 0.74, 95% CI: 0.56–0.97, *p* = 0.0066; PIAR: OR = 0.70, 95% CI: 0.53–0.94, *p* = 0.0271).

### 3.3 Pollen counts and PIAR symptoms in four areas

The primary clinical manifestations of rhinitis included sneezing, clear nasal discharge, nasal itching, and nasal congestion. Intermittent rhinitis was observed in 59.94% (SRAR), 56.28% (PDAR), and 55.91% (PIAR) of the patients, whereas seasonal rhinitis affected 59.35%, 65.05%, and 67.65% of the patients, respectively. Most cases were mild, with a disease duration of 1–3 years (Supplementary Table E2). Ocular symptoms, primarily itchy eyes, redness, and tearing, were generally mild. Additionally, nearly 60% of patients reported an itchy throat (Supplementary Table E3).

**Figure 2:**
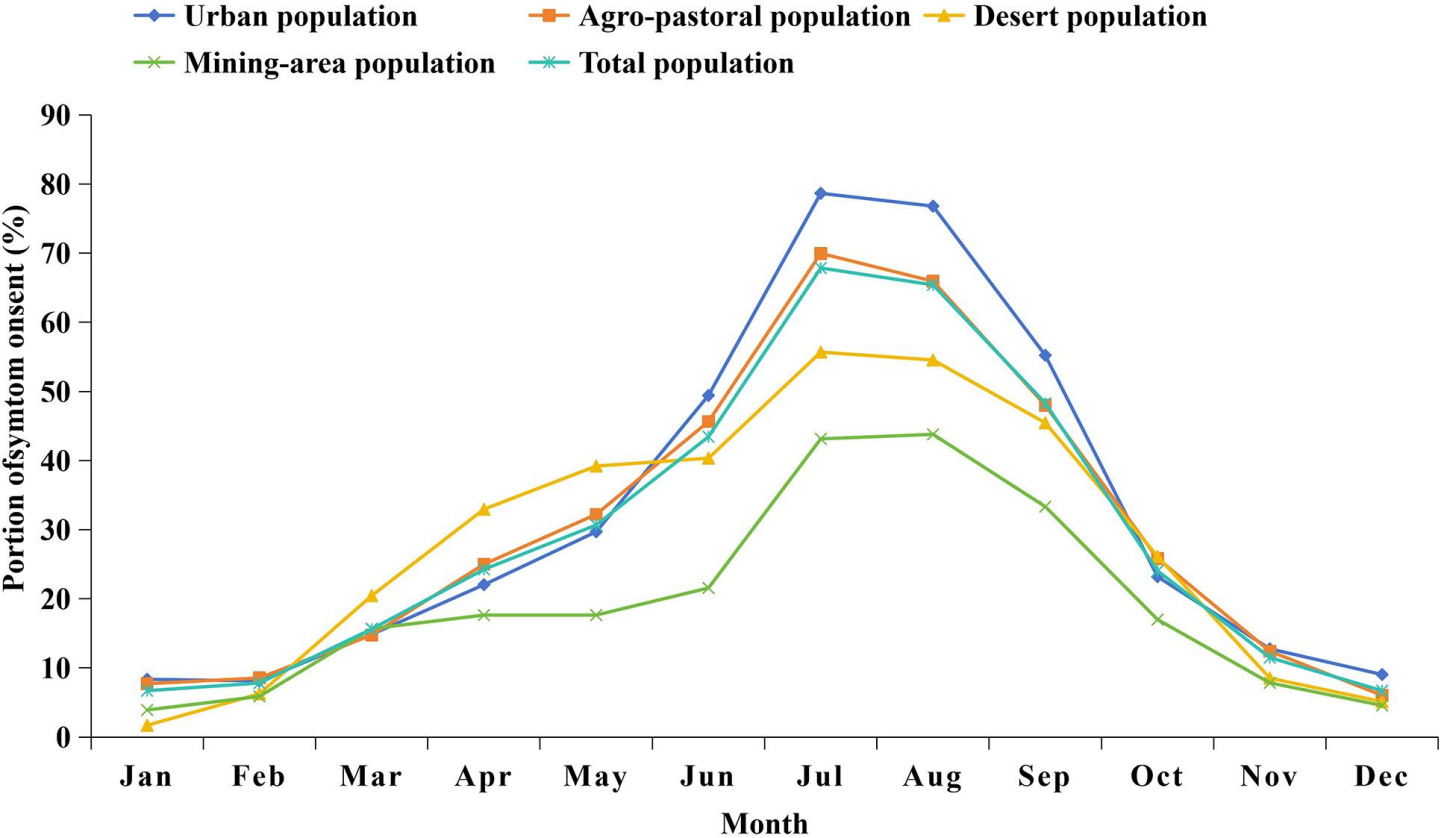
The incidence of symptom onset in urban, agro-pastoral, desert, and mining areas in 2023.

**Figure 3:**
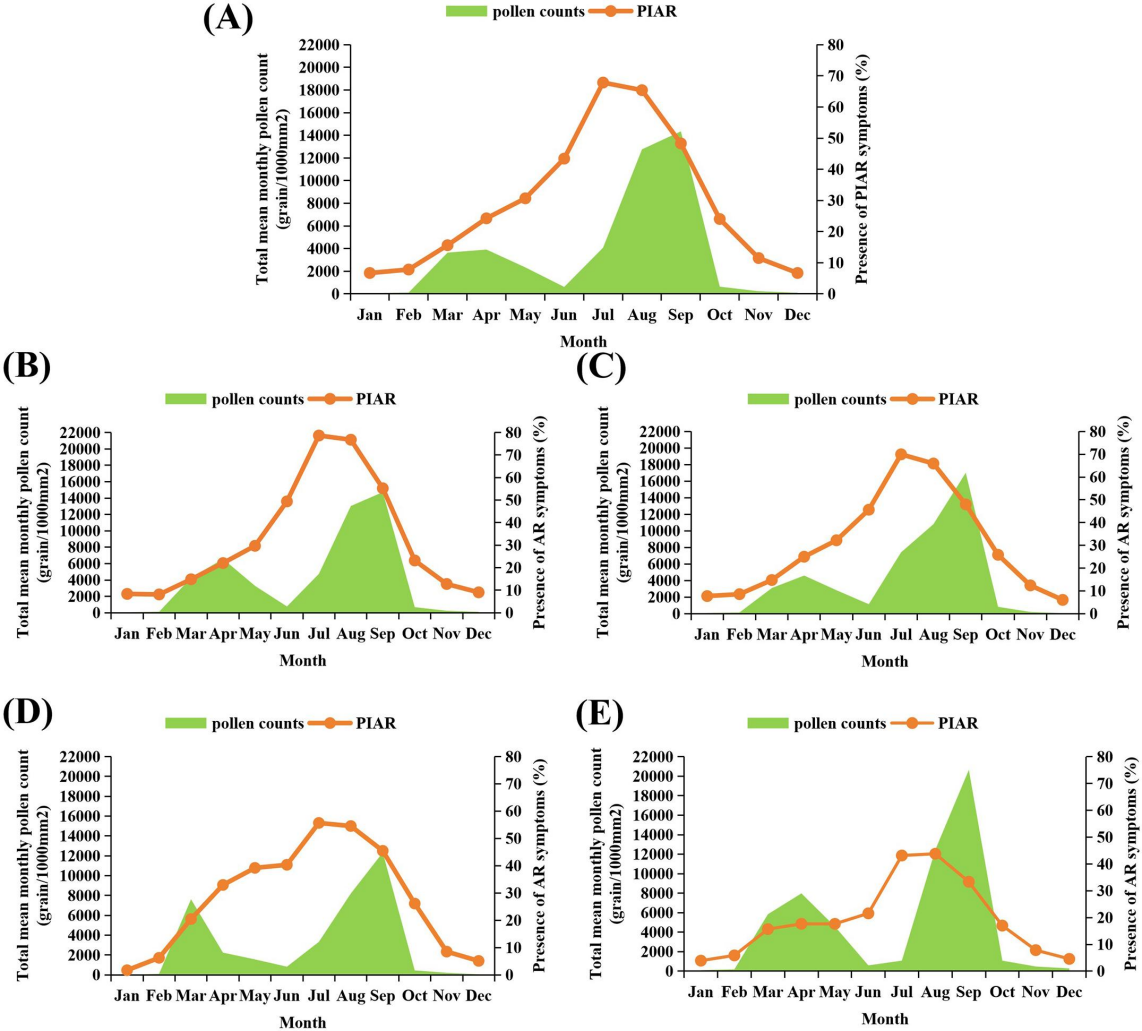
The mean total monthly pollen count and presence of symptoms for rhinitis for 12 months (2023) in the four study areas. **(A)** The overall data of the four areas. **(B–E)** The data of the four areas. **(B)** Urban area, **(C)** Agro-pastoral area, **(D)** Desert area, **(E)** Mining area.

**Figure 4:**
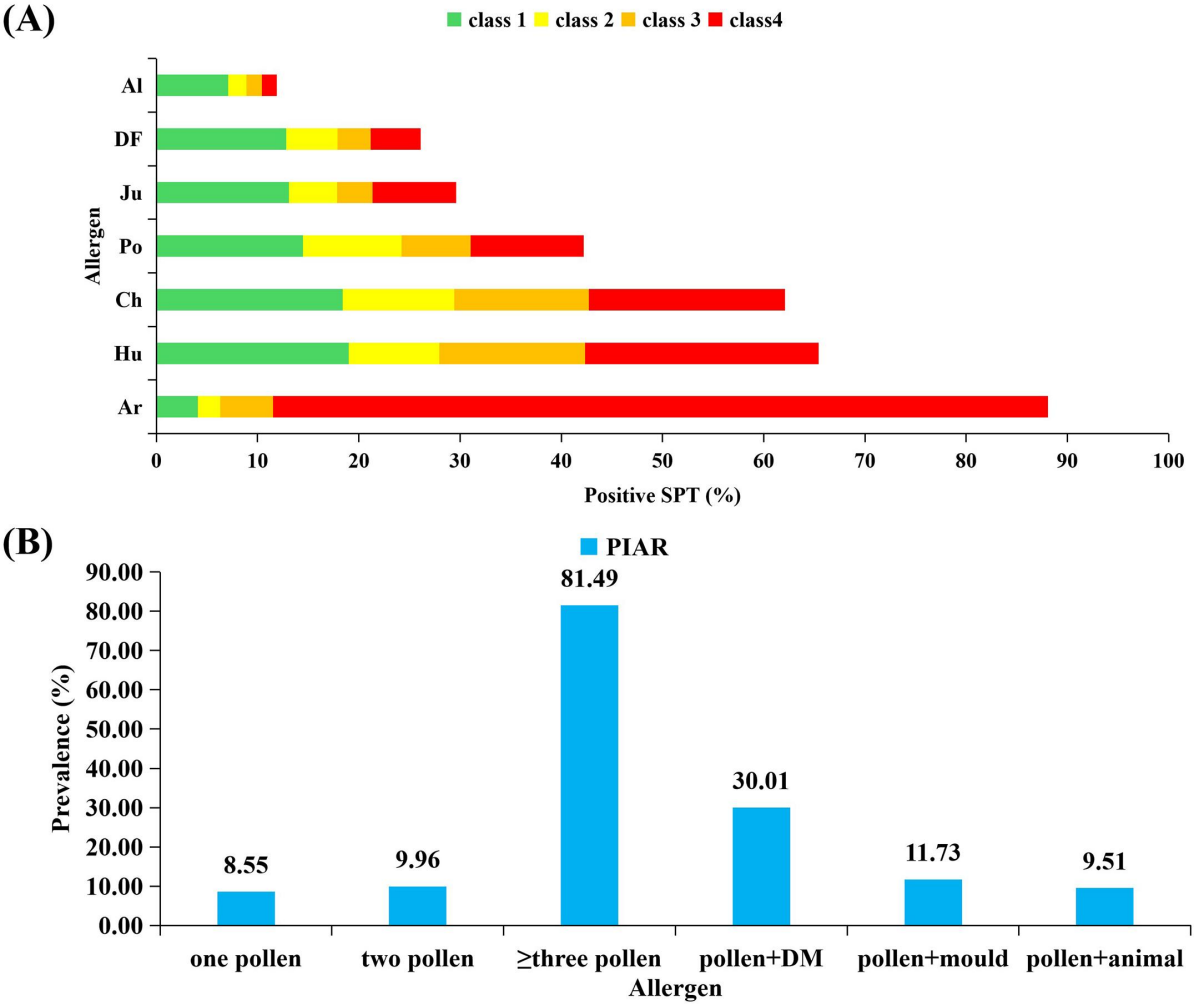
Allergen sensitization from skin prick test (SPT) in patients with PIAR. **(A)** Grading of positive SPT reactions to airborne pollen allergens, **(B)** The prevalence of different allergen combinations in patients with pollen-induced allergic rhinitis.

The prevalence rates of PIAR in urban areas, agropastoral areas, desert areas, and mining areas were 30.46%, 39.55%, 29.09%, and 19.72%, respectively (Figure 1, Table 3). The highest prevalence rate was recorded in agropastoral areas, and the lowest prevalence rate was recorded in mining areas. The peak symptom onset among patients with PIAR occurred in July and August, with urban areas having the highest incidence (79%) and mining areas having the lowest incidence (43%) (Figure 2). Pollen counts from January 1 to December 31, 2023 revealed two distinct pollen peaks in Ordos: one from March to April and another from August to September (Figure 3). The average total pollen count reached the maximum value in September (14,355 grains per 1,000 mm²) but exhibited significant regional differences. The urban, agropastoral, desert, and mining areas all exhibited two pollen peaks, with *Populus* pollen predominating in spring and *Artemisia* pollen in autumn. The mining area had the highest pollen count in September, reaching 20,651 grains per 1,000 mm². Although the mining area had the highest pollen count, it had the lowest incidence rate (43%) of PIAR symptoms (Figures 2, 3). This pollen–symptom paradox may be attributable to immune tolerance induced by chronic high-dose pollen exposure, which is consistent with prior reports of Treg-mediated suppression (13–15). Additionally, elevated PM2.5/PM10 levels in mining areas may facilitate pollen settling, while stricter occupational protective measures among residents could further reduce allergen exposure. *Artemisia* pollen was the most dominant in this region, accounting for 56.7% of the total, with a September peak of 12,082 grains per 1,000 mm² (Supplementary Figure E2). The SPT results revealed *Artemisia* (88.10%), *Humulus* (65.43%), and *Chenopodiaceae* (62.12%) as the primary allergenic pollens in PIAR (Figure 4). Interestingly, the highest symptom incidence occurred in July, when the pollen levels were substantially lower than the peak observed in September (Figure 3A).

**Table 3:** The prevalence of AR in the four areas.

Area	Total (*N* = 4,303) *n*(%)	SRAR		PDAR		PIAR	
		*n*	Prevalence (%)	*n*	Prevalence (%)	*n*	Prevalence (%)
Urban	1,415	713	50.39	477	33.71	431	30.46
Agro-pastoral	1,507	900	59.72	632	41.94	596	39.55
Desert	605	306	50.58	186	30.74	176	29.09
Mining	776	357	46.01	198	25.52	153	19.72

SRAR, self-reported allergic rhinitis; PDAR, physician-diagnosed allergic rhinitis; PIAR, pollen-induced allergic rhinitis.

### 3.4 Combined disease

Among the three types of AR, comorbidities, including skin conditions (such as eczema and dermatitis), were the most common. The prevalence rates of eczema for SRAR, PDAR, and PIAR were 25.37%, 28.21%, and 28.70%, respectively. Skin itching was reported by approximately 27% of patients (25.85% for SRAR, 27.23% for PDAR, and 27.10% for PIAR). Food allergy reactions were observed in approximately 23% of patients who had food allergies (19.30% for SRAR, 23.41% for PDAR, and 24.96% for PIAR), whereas approximately 10% of patients were affected by drug allergies (10.87% for SRAR, 10.23% for PDAR, and 10.36% for PIAR). Approximately 21% of patients had sinusitis (21.35% for SRAR, 21.57% for PDAR, and 21.02% for PIAR), with the primary symptoms being nasal congestion and purulent nasal discharge (anterior or posterior drainage into the throat). Chronic gastric disorders were observed in nearly 9% of the patients (10.71% for SRAR, 8.59% for PDAR, and 8.13% for PIAR), and asthma was diagnosed in nearly 12% of the patients (10.28% for SRAR, 12.66% for PDAR, and 13.05% for PIAR) (Supplementary Table E4).

### 3.5 Allergic sensitization from the skin prick test

The overall positive rate of SPT was 52.89%, with rates of 51.44% among adults and 54.98% among children. Pollen allergens had an SPT positivity rate of 49.52%. The three most common sensitizing pollen allergens among the patients with PIAR were *Artemisia* (88.10%), *Humulus* (65.43%), and *Chenopodiaceae* (62.12%). Notably, the *Artemisia* pollen had a rate of classC-4 positivity (≥2 cm in wheal diameter and the presence of pseudopods) as high as 76.58%. Additionally, 81.49% of the patients with PIAR were sensitized to three or more pollen types. Sensitivity to dust mites was observed in 30.01% of the patients with PIAR, to molds in 11.73%, and to both pollen and animal dander in 9.51% (Figure 4).

### 3.6 Cognitive and comprehensive prevention and control situation

Awareness of allergy prevention and control was suboptimal; 60% of the respondents obtained information about allergies from hospital physicians, 54% from family and friends, and 23% from organized prevention campaigns and control activities, online sources, or media channels. Approximately 62% of the respondents misidentified allergy symptoms for a cold (Supplementary Table E5).

### 3.7 Current treatment situation

In the treatment of patients with AR, predominant therapies, such as antihistamines and nasal corticosteroid sprays, allergen immunotherapy and monoclonal antibody therapy, are rarely performed, accounting for nearly 1% of treatments.

Among patients with SRAR, antihistamines (42.18%) and nasal corticosteroid sprays (27.15%) were most frequently used, followed by antiallergic eye drops (22.50%), nasal irrigation (18.98%), montelukast sodium (18.41%), traditional Chinese medicine (14.54%), nasal spray antihistamines (13.80%), antibiotic eye drops (12.08%), oral or intravenous antibiotics (9.89%), and oral corticosteroids (7.91%). Allergen immunotherapy and monoclonal antibody therapy were reported by only 0.79% of the patients (Supplementary Table E6).

Similar treatment patterns were observed in patients with PDAR and PIAR. Among patients with PDAR, antihistamines (49.16%), nasal corticosteroid sprays (31.55%), antiallergic eye drops (28.40%), nasal irrigation (22.84%), montelukast sodium (22.24%), traditional Chinese medicine (16.01%), nasal spray antihistamines (15.87%), antibiotic eye drops (14.67%), oral or intravenous antibiotics (10.38%), and oral corticosteroids (9.51%) were used. Allergen immunotherapy and monoclonal antibody therapy were used by 1.00% and 0.80% of the patients, respectively. Among patients with PIAR, antihistamines (50.88%), nasal corticosteroid sprays (32.74%), and antiallergic eye drops (30.09%) were the most prominent treatments, followed by nasal irrigation (23.45%), montelukast sodium (23.38%), traditional Chinese medicine (16.08%), nasal spray antihistamines (16.37%), antibiotic eye drops (15.63%), and oral or intravenous antibiotics (10.40%). Allergen immunotherapy and monoclonal antibody therapy were used by 1.11% and 0.88% of the patients, respectively.

## 4 Discussion

In this cross-sectional study conducted in a region with high pollen concentrations in northern China, for the first time, we investigated the prevalence of allergic diseases in Ordos by employing a combination of questionnaires, SPTs, and pollen monitoring. The prevalence rates of SRAR, PDAR, and PIAR were 52.89%, 34.70%, and 31.51%, respectively. Compared with Deng et al. (2019), who reported a PDAR prevalence of 22.36%, in the current study, we detected a markedly higher rate of 34.70%. However, differences in methodology, sampling, and diagnostic criteria between studies may reduce the credibility of the significant increase in prevalence. Moreover, the study by Deng et al. had a relatively small sample size, and it may not accurately reflect the current prevalence of AR in this region (8). Compared with the rates from our 2015 multicenter study conducted in six regions of Inner Mongolia (excluding Ordos), there were increases in prevalence rate of nearly 20% for SRAR in the current study (52.89% vs. 32.4%), nearly 16% for PDAR (34.70% vs. 18.8%), and nearly 13% for PIAR (31.51% vs. 18.5%). The prevalence rates of PDAR and PIAR in the Ordos region are close to those in Hohhot, indicating that the prevalence rates of AR and PIAR in Inner Mongolia, northern China, have reached high levels (7). However, in contrast to Hohhot, where these conditions occur in both spring and autumn, their incidence in the Ordos region is concentrated mainly in autumn, which is consistent with the fact that the concentration of autumn pollen in the Ordos region is higher than that in spring.

The elevated prevalence of AR in the Ordos region may be attributable to multiple factors. First, the increased public awareness of allergic diseases may have contributed to a notable increase in the prevalence of SRAR (52.89%). However, the lack of accurate scientific outreach may lead some patients to misjudge their condition, causing delays in standard diagnosis and treatment. The marked increase in the prevalence of PIAR may be linked to the significant increase in pollen exposure. *Artemisia* plants, widely used for windbreak and sand fixation in this region, account for 56.7% of the total pollen, reaching a peak at 12,082 grains per 1,000 mm² in September. High pollen levels can exacerbate PIAR symptoms (5, 16, 17). Climate change, coupled with low humidity and high wind speeds in this region, not only promotes pollen dispersion but also weakens the protective role of the respiratory tract mucosa (18–21). Additionally, extensive urban hard surfaces may hinder pollen deposition, elevating concentrations of suspended pollen and increasing inhalation risk (19, 22). Together, these factors explain the substantially higher AR prevalence in Ordos than in previous survey data from Inner Mongolia, highlighting the increased demand for public health resources for the prevention and control of this disease.

The peak onset age, 18–39 years, is consistent with our previously published research results in Inner Mongolia (23–25). AR prevalence is higher among the Mongolian ethnicity than among the Han ethnicity. These findings align with those reported by Deng et al. (8). However, earlier studies from our group reported no significant difference in AR prevalence between ethnic groups (5, 24). We found that compared with never-smokers, ever-smokers had a greater risk of SRAR, whereas current smokers demonstrated a lower risk of PDAR/PIAR. This pattern aligns with Swedish data showing reduced AR prevalence in male smokers but conflicts with the findings of a large French survey indicating elevated AR and hay fever risk in adolescents (26, 27). These discrepancies may be explained by differential immunological effects: current smoking suppresses Th2 responses, reduces IgE levels, and inhibits IL-33 release, potentially alleviating AR symptoms (28). Conversely, smoking cessation reverses this suppression, reactivating Th2 pathways and upregulating IL-33/TSLP to induce mucosal inflammation and rebound allergic susceptibility in former smokers (29).

We are one of the few research teams that have investigated the prevalence of PIAR and the incidence of PIAR symptoms and have monitored pollen concentrations by classifying areas into urban, agricultural and pastoral, desert, and mining regions. The prevalence of PIAR was the highest in agropastoral areas (39.55%) and the lowest in mining areas (19.72%). For patients with PIAR, symptom onset peaked in July and August, and the highest incidence rate was noted in urban areas (79%) and the lowest in mining areas (43%). The high prevalence of AR in agropastoral areas is closely associated with prolonged outdoor exposure during the day and high vegetation diversity. Urban residents, mainly civil servants and commercial or service sector employees, typically work in enclosed office settings. It is hypothesized that insufficient ventilation may promote the accumulation of indoor allergens (e.g., dust mites and mold), thereby increasing symptom rates in urban areas. In contrast, workers in mines often use respiratory protection in underground or open-pit settings, leading to a marked reduction in allergen exposure and thus lowering the prevalence of AR. These findings indicate that better personal protection and reduced exposure to allergens can effectively mitigate the risk for disease in high-incidence areas, highlighting their importance in AR prevention and control.

In Ordos, the average total pollen count peaked in April and September, reaching a maximum of 14,355 grains per 1,000 mm² in September, but with significant regional variations. The urban, agropastoral, desert, and mining areas all exhibited two pollen peaks in spring and autumn. *Populus* pollen predominated in spring, whereas *Artemisia* pollen was dominant in autumn. Notably, the highest pollen counts were recorded in the mining area in September, whereas the lowest were recorded in the desert area. Pollen monitoring data indicated that *Artemisia* pollen accounted for the greatest proportion (56.7%) of the total pollen, with peak concentrations of 12,082 grains per 1,000 mm² in September. SPTs revealed that the primary allergen pollens for patients with PIAR were *Artemisia* (88.10%), *Humulus* (65.43%), and *Chenopodiaceae* (62.12%).

Previous studies have suggested that long-term environmental exposure may promote immune tolerance through mechanisms such as regulatory T cells (Tregs) (13–15). Consistent with this possibility, our study revealed that the mining area presented the lowest prevalence and symptom incidence of pollen-induced allergic rhinitis (PIAR) during the pollen season, despite having the highest pollen concentration. This may be explained by the large-scale planting of *Artemisia* for vegetation restoration and ecological reconstruction after mining activities; prolonged exposure to high levels of pollen may induce tolerance to specific *Artemisia* allergens in this region. High PM2.5/PM10 concentrations in mining areas may adhere to pollen surfaces, reducing the airborne time of pollen and accelerating the settling speed of pollen (30, 31). In addition, residents in mining areas may have higher occupational health awareness and adopt stricter protective measures (e.g., wearing masks and protective equipment), which contribute to the lower prevalence and symptom incidence of PIAR. Furthermore, our study revealed that approximately 62% of the respondents misidentified allergic symptoms as cold symptoms, indicating the need for public education campaigns in this region to improve awareness of allergic rhinitis (AR), reduce the risk of misdiagnosis, and promote timely and accurate diagnosis and treatment.

Nevertheless, our study has several limitations. First, the cross-sectional design cannot establish causality, and the retrospective risk factor analysis may have introduced bias. Second, the SPT allergen panels in children were less comprehensive than those in adults were, which could limit the completeness and accuracy of the study findings related to pediatric allergen sensitization. Third, environmental parameters, including temperature and humidity, were not analyzed. Future studies will need to examine larger cohorts employing standardized survey forms and assess a larger number of confounding risk parameters (i.e., endotoxins, parasites, and air pollution) in an attempt to better understand the prevalence, difference, and relationship between AR and asthma for better generalizability.

## 5 Conclusion

In this study, the prevalence, risk factors, and treatment conditions of AR and PIAR in the high-pollen grassland regions of northern China were comprehensively investigated. AR prevalence in this region is markedly high, with *Artemisia* pollen identified as the predominant allergen and a key risk factor. A distinct aspect of this research is its stratification of the grassland region into urban, agricultural and pastoral, desert, and mining areas, facilitating an assessment of the relative effects of pollen concentrations and symptom severity across different environments. These findings provide a basis for developing long-term, integrated prevention and control strategies, with implications not only for China but also for national or regional policies in other grassland ecosystems with similar characteristics.

## Data Availability

The raw data supporting the conclusions of this article will be made available by the authors, without undue reservation.
