# Supplementary material for: Pollen-induced allergic rhinitis in the central region of Inner Mongolia, China: prevalence, risk factors, and regional characteristics
Source: Front Allergy. 2026 May 11;7:1800197. doi: 10.3389/falgy.2026.1800197 (PMC13199316; doi:10.3389/falgy.2026.1800197)
Supplement: Supplementary file 1 [file Image1.pdf]

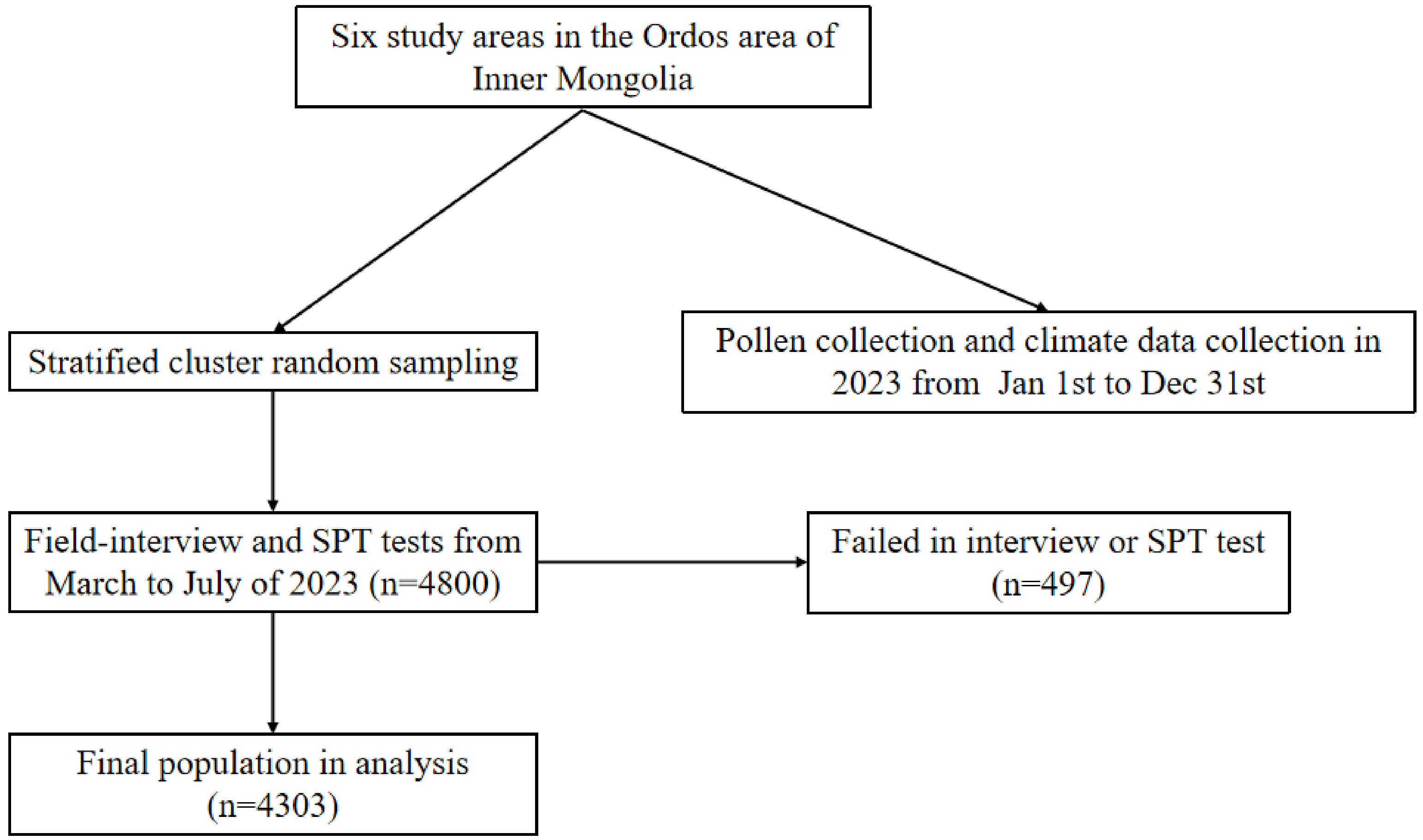

Fig. E1 Flow chart of the study design.

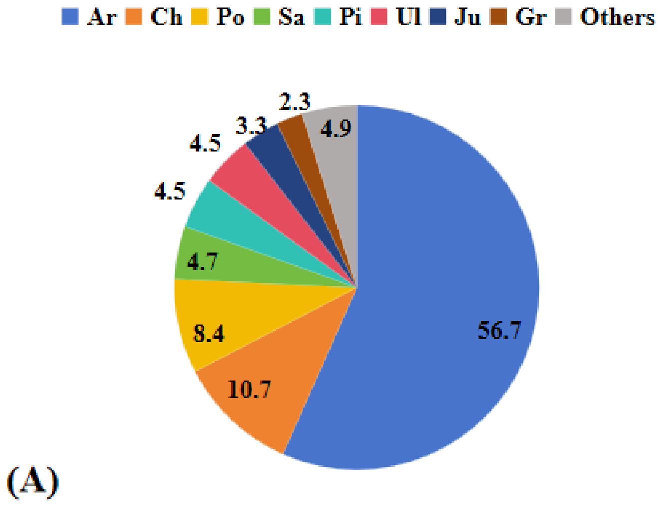

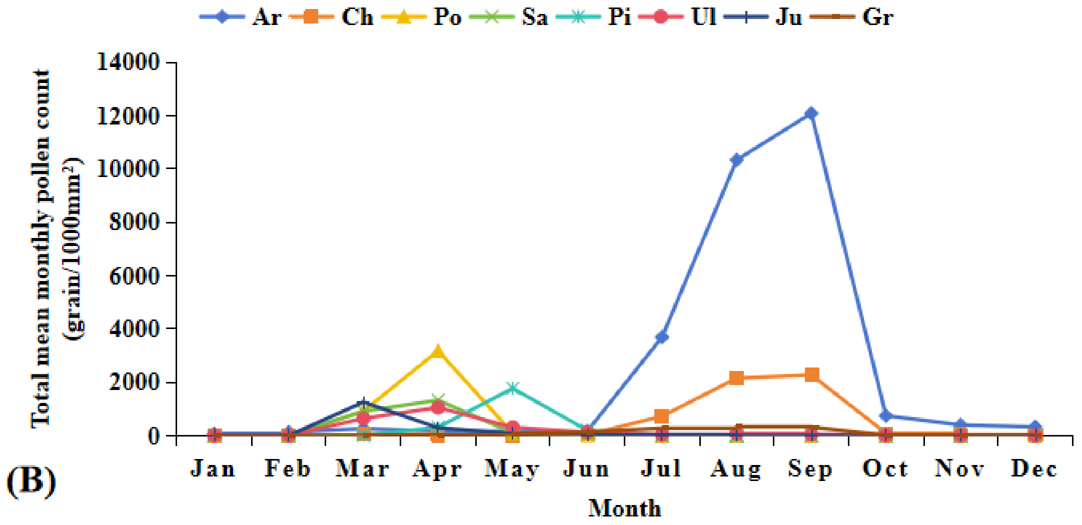

Fig. E2 (A) Proportion of different pollen types (%). (B) Pollen dispersal for 12 month in 2023.
