# Supplementary material for: Pollen-induced allergic rhinitis in the central region of Inner Mongolia, China: prevalence, risk factors, and regional characteristics
Source: Front Allergy. 2026 May 11;7:1800197. doi: 10.3389/falgy.2026.1800197 (PMC13199316; doi:10.3389/falgy.2026.1800197)
Supplement: Supplementary file 2 [file Table1.docx]

**TABLE E1. Characteristics of subjects in the SRAR, PDAR, and PIAR groups**

| **Characters** | **Total  (N=4303)  n(%)** | **SRAR^^[[1]](#footnote-1)^†^** | | | | | **PDAR^^[[2]](#footnote-2)^‡^** | | | | | **PIAR^^[[3]](#footnote-3)^§^** | | | | |  |
| --- | --- | --- | --- | --- | --- | --- | --- | --- | --- | --- | --- | --- | --- | --- | --- | --- | --- |
|  |  | **P(95%CI)** | **Yes (n=2276)** | **No (n=2027)** | **χ^2^** | ***P*** | **P(95%CI)** | **Yes (n=1493)** | **No (n=2810)** | **χ^2^** | ***P*** | **P(95%CI)** | **Yes (n=1356)** | **No (n=2947)** | **χ^2^** | ***P*** |  |
|  |  |  |  |  |  |  |  |  |  |  |  |  |  |  |  |  |  |
|  |  |  |  |  |  |  |  |  |  |  |  |  |  |  |  |  |  |
| Degree of education |  |  |  |  | 89.77 | <0.0001 |  |  |  | 102.28 | <0.0001 |  |  |  | 94.38 | <0.0001 |  |
| Elementary school | 1265 (29.40) | 44.82 (42.06,47.61) | 567 (24.91) | 698 (34.44) | . | . | 28.14 (25.68,30.71) | 356 (23.84) | 909 (32.35) | . | . | 25.38 (23.00,27.87) | 321 (23.67) | 944 (32.03) | . | . |  |
| Middle school | 1688 (39.23) | 50.95 (48.53,53.36) | 860 (37.79) | 828 (40.85) | . | . | 31.04 (28.84,33.31) | 524 (35.10) | 1164 (41.42) | . | . | 28.08 (25.95,30.29) | 474 (34.96) | 1214 (41.19) | . | . |  |
| University and above | 1350 (31.37) | 62.89 (60.25,65.47) | 849 (37.30) | 501 (24.72) | . | . | 45.41 (42.73,48.11) | 613 (41.06) | 737 (26.23) | . | . | 41.56 (38.91,44.24) | 561 (41.37) | 789 (26.77) | . | . |  |
| Annual income(CNY, ×104) |  |  |  |  | 8.15 | 0.017 |  |  |  | 42.26 | <0.0001 |  |  |  | 41.13 | <0.0001 |  |
| <5 | 600 (13.94) | 49.83 (45.76,53.91) | 299 (13.14) | 301 (14.85) | . | . | 26.33 (22.85,30.05) | 158 (10.58) | 442 (15.73) | . | . | 23.17 (19.85,26.75) | 139 (10.25) | 461 (15.64) | . | . | . |
| 5~10 | 1850 (42.99) | 51.46 (49.15,53.76) | 952 (41.83) | 898 (44.30) | . | . | 32.49 (30.35,34.67) | 601 (40.25) | 1249 (44.45) | . | . | 29.57 (27.49,31.71) | 547 (40.34) | 1303 (44.21) | . | . | . |
| >10 | 1853 (43.06) | 55.32 (53.02,57.60) | 1025 (45.04) | 828 (40.85) | . | . | 39.61 (37.38,41.88) | 734 (49.16) | 1119 (39.82) | . | . | 36.16 (33.97,38.39) | 670 (49.41) | 1183 (40.14) | . | . | . |
| Family history |  |  |  |  | 114.20 | <0.0001 |  |  |  | 92.87 | <0.0001 |  |  |  | 96.31 | <0.0001 |  |
| No | 2447 (56.87) | 45.81 (43.82,47.81) | 1121 (49.25) | 1326 (65.42) | . | . | 28.61 (26.82,30.44) | 700 (46.89) | 1747 (62.17) |  |  | 25.46 (23.74,27.23) | 623 (45.94) | 1824 (61.89) |  |  |  |
| Yes | 1856 (43.13) | 62.23 (59.98,64.44) | 1155 (50.75) | 701 (34.58) | . | . | 42.73 (40.46,45.01) | 793 (53.11) | 1063 (37.83) |  |  | 39.49 (37.26,41.76) | 733 (54.06) | 1123 (38.11) |  |  |  |
| Keeping pets |  |  |  |  | 5.50 | 0.0191 |  |  |  | 10.78 | 0.001 |  |  |  | 9.33 | 0.0022 |  |
| No | 3597 (83.59) | 53.68 (52.04,55.32) | 1931 (84.84) | 1666 (82.19) | . | . | 35.75 (34.18,37.34) | 1286 (86.14) | 2311 (82.24) |  |  | 32.47 (30.94,34.03) | 1168 (86.14) | 2429 (82.42) |  |  |  |
| Yes | 706 (16.41) | 48.87 (45.12,52.62) | 345 (15.16) | 361 (17.81) | . | . | 29.32 (25.98,32.83) | 207 (13.86) | 499 (17.76) |  |  | 26.63 (23.40,30.05) | 188 (13.86) | 518 (17.58) |  |  |  |
| Residential environment |  |  |  |  | 31.36 | <0.0001 |  |  |  | 50.66 | <0.0001 |  |  |  | 50.78 | <0.0001 |  |
| Building | 3422 (79.53) | 55.06 (53.37,56.73) | 1884 (82.78) | 1538 (75.88) | . |  | 37.32 (35.69,38.96) | 1277 (85.53) | 2145 (76.33) | . | . | 34.07 (32.49,35.69) | 1166 (85.99) | 2256 (76.55) |  |  |  |
| Bungalow | 881 (20.47) | 44.49 (41.18,47.85) | 392 (17.22) | 489 (24.12) | . |  | 24.52 (21.71,27.50) | 216 (14.47) | 665 (23.67) | . | . | 21.57 (18.89,24.43) | 190 (14.01) | 691 (23.45) |  |  |  |
| Work/study environment |  |  |  |  | 0.01 | 0.9212 |  |  |  | 11.78 | 0.0006 |  |  |  | 20.61 | <0.0001 |  |
| Building | 3466 (80.55) | 52.86 (51.18,54.53) | 1832 (80.49) | 1634 (80.61) | . |  | 35.92 (34.32,37.54) | 1245 (83.39) | 2221 (79.04) | . | . | 33.09 (31.53,34.69) | 1147 (84.59) | 2319 (78.69) |  |  |  |
| Bungalow | 837 (19.45) | 53.05 (49.60,56.47) | 444 (19.51) | 393 (19.39) | . |  | 29.63 (26.55,32.85) | 248 (16.61) | 589 (20.96) | . | . | 24.97 (22.07,28.05) | 209 (15.41) | 628 (21.31) |  |  |  |
| Outdoor activity (hours/day) |  |  |  |  | 3.97 | 0.2649 |  |  |  | 0.67 | 0.8814 |  |  |  | 1.52 | 0.6786 |  |
| ＜1 h | 1134 (26.62) | 54.67 (51.72,57.60) | 620 (27.53) | 514 (25.60) | . |  | 35.10 (32.32,37.95) | 398 (26.95) | 736 (26.45) | . | . | 31.75 (29.04,34.54) | 360 (26.83) | 774 (26.53) |  |  |  |
| 1-2 h | 1765 (41.43) | 51.56 (49.20,53.91) | 910 (40.41) | 855 (42.58) | . |  | 34.45 (32.23,36.72) | 608 (41.16) | 1157 (41.57) | . | . | 31.44 (29.28,33.67) | 555 (41.36) | 1210 (41.47) |  |  |  |
| 2-3 h | 649 (15.23) | 51.46 (47.54,55.37) | 334 (14.83) | 315 (15.69) | . |  | 35.59 (31.91,39.41) | 231 (15.64) | 418 (15.02) | . | . | 32.97 (29.36,36.74) | 214 (15.95) | 435 (14.91) |  |  |  |
| ≥3 h | 712 (16.71) | 54.49 (50.75,58.20) | 388 (17.23) | 324 (16.14) | . |  | 33.71 (30.24,37.31) | 240 (16.25) | 472 (16.96) | . | . | 29.92 (26.57,33.43) | 213 (15.87) | 499 (17.10) |  |  |  |
| Special occupation(≥18years) |  |  |  |  | 1.78 | 0.1827 |  |  |  | 1.00 | 0.3168 |  |  |  | 2.24 | 0.1343 |  |
| No | 2382 (93.89) | 58.35 (56.34,60.34) | 1390 (94.43) | 992 (93.15) | . |  | 36.9 (34.96,38.88) | 879 (94.52) | 1503 (93.53) |  |  | 32.91 (31.03,34.84) | 784 (94.92) | 1598 (93.40) |  |  |  |
| Yes | 155 (6.11) | 52.9 (44.73,60.96) | 82 (5.57) | 73 (6.85) | . |  | 32.90 (25.58,40.90) | 51 (5.48) | 104 (6.47) |  |  | 27.10 (20.28,34.81) | 42 (5.08) | 113 (6.60) |  |  |  |
| Marital status (≥18years) |  |  |  |  | 2.49 | 0.1144 |  |  |  | 3.16 | 0.0756 |  |  |  | 4.24 | 0.0395 |  |
| Single | 441 (17.38) | 54.65 (49.87,59.36) | 241 (16.37) | 200 (18.78) | . |  | 40.36 (35.75,45.11) | 178 (19.14) | 263 (16.37) | . | . | 36.73 (32.22,41.42) | 162 (19.61) | 279 (16.31) |  |  |  |
| Married | 2096 (82.62) | 58.73 (56.59,60.85) | 1231 (83.63) | 865 (81.22) | . |  | 35.88 (33.82,37.97) | 752 (80.86) | 1344 (83.63) | . | . | 31.68 (29.69,33.72) | 664 (80.39) | 1432 (83.69) |  |  |  |
| BMI (≥18years) |  |  |  |  | 1.83 | 0.6074 |  |  |  | 4.84 | 0.184 |  |  |  | 5.49 | 0.1394 |  |
| Underweight | 56 (2.21) | 50.00 (36.34,63.66) | 28 (1.90) | 28 (2.63) | . |  | 32.14 (20.29,45.96) | 18 (1.94) | 38 (2.36) | . | . | 28.57 (17.30,42.21) | 16 (1.94) | 40 (2.34) |  |  |  |
| Normal weight | 812 (32.02) | 58.00 (54.52,61.43) | 471 (32.02) | 341 (32.02) | . |  | 38.42 (35.06,41.87) | 312 (33.58) | 500 (31.11) | . | . | 34.48 (31.21,37.87) | 280 (33.94) | 532 (31.09) |  |  |  |
| Overweight | 1039 (40.97) | 57.75 (54.68,60.77) | 600 (40.79) | 439 (41.22) | . |  | 34.36 (31.47,37.34) | 357 (38.43) | 682 (42.44) | . | . | 30.13 (27.35,33.02) | 313 (37.94) | 726 (42.43) |  |  |  |
| Obesity | 629 (24.80) | 59.14 (55.18,63.01) | 372 (25.29) | 257 (24.13) | . |  | 38.47 (34.65,42.40) | 242 (26.05) | 387 (24.08) | . | . | 34.34 (30.63,38.20) | 216 (26.18) | 413 (24.14) |  |  |  |
| Abdominal obesity(≥18years) |  |  |  |  | 0.04 | 0.8497 |  |  |  | 3.71 | 0.054 |  |  |  | 4.61 | 0.0318 |  |
| Normal | 1145 (45.13) | 57.82 (54.90,60.70) | 662 (44.97) | 483 (45.35) | . |  | 38.69 (35.86,41.58) | 443 (47.63) | 702 (43.68) |  |  | 34.76 (32.00,37.60) | 398 (48.18) | 747 (43.66) |  |  |  |
| Abdominal obesity | 1392 (54.87) | 58.19 (55.55,60.80) | 810 (55.03) | 582 (54.65) | . |  | 34.99 (32.48,37.56) | 487 (52.37) | 905 (56.32) |  |  | 30.75 (28.33,33.25) | 428 (51.82) | 964 (56.34) |  |  |  |
| Smoking (≥18years) |  |  |  |  | 10.71 | 0.0047 |  |  |  | 7.96 | 0.0187 |  |  |  | 8.91 | 0.0116 |  |
| Never | 1939 (76.88) | 58.07 (55.84,60.28) | 1126 (76.97) | 813 (76.77) | . |  | 37.80 (35.64,40.00) | 733 (78.99) | 1206 (75.66) | . | . | 34.14 (32.03,36.30) | 662 (80.34) | 1277 (75.21) |  |  |  |
| Current | 460 (18.24) | 54.35 (49.67,58.97) | 250 (17.09) | 210 (19.83) | . |  | 31.30 (27.09,35.76) | 144 (15.52) | 316 (19.82) | . | . | 26.96 (22.95,31.26) | 124 (15.05) | 336 (19.79) |  |  |  |
| Ever | 123 (4.88) | 70.73 (61.85,78.59) | 87 (5.95) | 36 (3.40) | . |  | 41.46 (32.65,50.69) | 51 (5.50) | 72 (4.52) | . | . | 30.89 (22.88,39.86) | 38 (4.61) | 85 (5.01) |  |  |  |
| Term Birth(<18years) |  |  |  |  | 0.02 | 0.8986 |  |  |  | 0.32 | 0.5746 |  |  |  | 0.82 | 0.3643 |  |
| No | 50 (3.00) | 46.00 (31.81,60.68) | 23 (3.05) | 27 (2.95) | . | . | 28.00 (16.23,42.49) | 14 (2.65) | 36 (3.16) | . | . | 24.00 (13.06,38.17) | 12 (2.41) | 38 (3.24) |  |  |  |
| Yes | 1619 (97.00) | 45.09 (42.65,47.55) | 730 (96.95) | 889 (97.05) | . | . | 31.75 (29.48,34.08) | 514 (97.35) | 1105 (96.84) | . | . | 29.96 (27.73,32.25) | 485 (97.59) | 1134 (96.76) |  |  |  |
| Premature Birth(<18years) |  |  |  |  | 0.98 | 0.3233 |  |  |  | 0.19 | 0.6626 |  |  |  | 0.05 | 0.8257 |  |
| No | 1702 (96.38) | 45.30 (42.92,47.70) | 771 (95.90) | 931 (96.78) | . | . | 31.79 (29.58,34.06) | 541 (96.09) | 1161 (96.51) | . | . | 29.96 (27.80,32.20) | 510 (96.23) | 1192 (96.44) |  |  |  |
| Yes | 64 (3.62) | 51.56 (38.73,64.25) | 33 (4.10) | 31 (3.22) | . | . | 34.38 (22.95,47.30) | 22 (3.91) | 42 (3.49) | . | . | 31.25 (20.24,44.06) | 20 (3.77) | 44 (3.56) |  |  |  |
| Postterm Birth(<18years) |  |  |  |  | 2.55 | 0.1106 |  |  |  | 5.74 | 0.0166 |  |  |  | 4.30 | 0.0382 |  |
| No | 1749 (99.04) | 45.34 (42.99,47.71) | 793 (98.63) | 956 (99.38) | . | . | 31.62 (29.44,33.86) | 553 (98.22) | 1196 (99.42) | . | . | 29.79 (27.65,31.99) | 521 (98.3) | 1228 (99.35) |  |  |  |
| Yes | 17 (0.96) | 64.71 (38.33,85.79) | 11 (1.37) | 6 (0.62) | . | . | 58.82 (32.92,81.56) | 10 (1.78) | 7 (0.58) | . | . | 52.94 (27.81,77.02) | 9 (1.70) | 8 (0.65) |  |  |  |
| Delivery mode(<18years) |  |  |  |  | 16.65 | <0.0001 |  |  |  | 40.82 | <0.0001 |  |  |  | 41.26 | <0.0001 |  |
| Natural delivery | 1300 (74.58) | 42.77 (40.06,45.51) | 556 (69.94) | 744 (78.48) | . | . | 27.85 (25.42,30.37) | 362 (64.87) | 938 (79.16) | . | . | 26.00 (23.63,28.48) | 338 (64.38) | 962 (78.98) |  |  |  |
| Caesarean section | 443 (25.42) | 53.95 (49.18,58.66) | 239 (30.06) | 204 (21.52) | . | . | 44.24 (39.56,49.01) | 196 (35.13) | 247 (20.84) | . | . | 42.21 (37.57,46.96) | 187 (35.62) | 256 (21.02) |  |  |  |
| Type of feeding (<18years) |  |  |  |  | 6.22 | 0.0447 |  |  |  | 2.59 | 0.2736 |  |  |  | 1.97 | 0.3735 |  |
| Breastfeeding | 608 (35.04) | 49.34 (45.30,53.39) | 300 (37.88) | 308 (32.66) | . | . | 34.38 (30.6,38.30) | 209 (37.59) | 399 (33.84) |  |  | 32.24 (28.53,36.11) | 196 (37.48) | 412 (33.99) |  |  |  |
| Non breastfeeding | 125 (7.20) | 48.00 (38.98,57.11) | 60 (7.58) | 65 (6.89) | . | . | 32.80 (24.67,41.77) | 41 (7.37) | 84 (7.12) |  |  | 29.60 (21.77,38.42) | 37 (7.07) | 88 (7.26) |  |  |  |
| Mixed type | 1002 (57.75) | 43.11 (40.02,46.25) | 432 (54.55) | 570 (60.45) | . | . | 30.54 (27.70,33.49) | 306 (55.04) | 696 (59.03) |  |  | 28.94 (26.15,31.86) | 290 (55.45) | 712 (58.75) |  |  |  |

**TABLE E2. Clinical characteristics of the SRAR, PDAR, and PIAR groups**

| **Variable** |  | **SRAR** | | **PDAR** | | **PIAR** | |
| --- | --- | --- | --- | --- | --- | --- | --- |
|  |  | Number (n=2276) | Percentage (%) | Number (n=1493) | Percentage (%) | Number (n=1356) | Percentage (%) |
| **Duration of illness** | Intermittent | 1001 | 59.94 | 632 | 56.28 | 572 | 55.91 |
|  | Persistent | 669 | 40.06 | 491 | 43.72 | 451 | 44.09 |
|  | Total | 1670 | 100.00 | 1123 | 100.00 | 1023 | 100.00 |
| **Seasonal variation of illness** | Perennial without seasonal variation | 443 | 21.41 | 205 | 14.62 | 162 | 12.60 |
|  | Perennial with seasonal exacerbations | 398 | 19.24 | 285 | 20.33 | 254 | 19.75 |
|  | Seasonal | 1228 | 59.35 | 912 | 65.05 | 870 | 67.65 |
|  | Total | 2069 | 100.00 | 1402 | 100.00 | 1286 | 100.00 |
| **Disease course (year)** | <1 y | 122 | 5.70 | 55 | 3.82 | 49 | 3.71 |
|  | 1-3 y | 661 | 30.90 | 429 | 29.81 | 382 | 28.96 |
|  | 4-5 y | 466 | 21.79 | 320 | 22.24 | 295 | 22.37 |
|  | 6-10 y | 429 | 20.06 | 320 | 22.24 | 304 | 23.05 |
|  | >10 y | 461 | 21.55 | 315 | 21.89 | 289 | 21.91 |
|  | Total | 2139 | 100.00 | 1439 | 100.00 | 1319 | 100.00 |

**TABLE E3. Clinical symptoms of SRAR, PDAR, and PIAR groups**

| **Symptom** | | **SRAR** | | | | | | **PDAR** | | | | | **PIAR** | | | | | |
| --- | --- | --- | --- | --- | --- | --- | --- | --- | --- | --- | --- | --- | --- | --- | --- | --- | --- | --- |
|  |  | **No** | **Mild** | **Moderate** | **Severe** | **Total** | **No** | | **Mild** | **Moderate** | **Severe** | **Total** | | **No** | **Mild** | **Moderate** | **Severe** | **Total** |
|  |  | **(%)** | **(%)** | **(%)** | **(%)** | **(%)** | **(%)** | | **(%)** | **(%)** | **(%)** | **(%)** | | **(%)** | **(%)** | **(%)** | **(%)** | **(%)** |
| **Symptoms of rhinitis and rhinoconjunctivitis** | Itchy nose | 284 (12.48) | 820 (41.71) | 611 (31.08) | 534 (27.16) | 1992 (87.52) | 160 (10.72) | | 497 (37.62) | 438 (33.16) | 385 (29.14) | 1333 (89.28) | | 139 (10.25) | 436 (36.18) | 407 (33.78) | 361 (29.96) | 1217 (89.75) |
|  | Sneezing | 132 (5.80) | 879 (41.42) | 635 (29.92) | 608 (28.65) | 2144 (94.20) | 79 (5.29) | | 511 (36.45) | 464 (33.10) | 427 (30.46) | 1414 (94.71) | | 71 (5.24) | 443 (34.8) | 431 (33.86) | 399 (31.34) | 1285 (94.76) |
|  | Runny nose | 344 (15.11) | 769 (40.73) | 602 (31.89) | 517 (27.38) | 1932 (84.89) | 203 (13.6) | | 468 (37.03) | 433 (34.26) | 363 (28.72) | 1290 (86.4) | | 180 (13.27) | 419 (36.37) | 389 (33.77) | 344 (29.86) | 1176 (86.73) |
|  | Nasal obstruction | 399 (17.53) | 648 (35.31) | 572 (31.17) | 614 (33.46) | 1877 (82.47) | 204 (13.66) | | 404 (32.04) | 411 (32.59) | 445 (35.29) | 1289 (86.34) | | 172 (12.68) | 364 (31.46) | 375 (32.41) | 417 (36.04) | 1184 (87.32) |
|  | Itchy throat | 970 (42.62) | 749 (58.61) | 305 (23.87) | 224 (17.53) | 1306 (57.38) | 602 (40.32) | | 468 (53.67) | 227 (26.03) | 177 (20.30) | 891 (59.68) | | 548 (40.41) | 409 (51.77) | 211 (26.71) | 170 (21.52) | 808 (59.59) |
|  | Itchy eyes | 535 (23.51) | 772 (45.44) | 447 (26.31) | 480 (28.25) | 1741 (76.49) | 247 (16.54) | | 466 (38.42) | 346 (28.52) | 401 (33.06) | 1246 (83.46) | | 211 (15.56) | 405 (36.42) | 323 (29.05) | 384 (34.53) | 1145 (84.44) |
|  | Bloodshot eyes | 923 (40.55) | 710 (53.95) | 362 (27.51) | 244 (18.54) | 1353 (59.45) | 531 (35.57) | | 455 (48.35) | 290 (30.82) | 196 (20.83) | 962 (64.43) | | 471 (34.73) | 401 (46.25) | 277 (31.95) | 189 (21.80) | 885 (65.27) |
|  | Shedding tears | 840 (36.91) | 807 (57.93) | 356 (25.56) | 230 (16.51) | 1436 (63.09) | 528 (35.37) | | 521 (55.48) | 248 (26.41) | 170 (18.10) | 965 (64.63) | | 481 (35.47) | 457 (53.83) | 231 (27.21) | 161 (18.96) | 875 (64.53) |
| **Symptoms of sinusitis** | Nasal obstruction | 1661 (72.98) | 240 (39.02) | 155 (25.20) | 220 (35.77) | 615 (27.02) | 1087 (72.81) | | 145 (35.71) | 111 (27.34) | 150 (36.95) | 406 (27.19) | | 990 (73.01) | 129 (35.25) | 94 (25.68) | 143 (39.07) | 366 (26.99) |
|  | Purulent nasal discharge (either flowing forward or draining backward into the pharynx) | 1873 (82.29) | 218 (54.09) | 114 (28.29) | 71 (17.62) | 403 (17.71) | 1230 (82.38) | | 137 (52.09) | 78 (29.66) | 48 (18.25) | 263 (17.62) | | 1124 (82.89) | 121 (52.16) | 69 (29.74) | 42 (18.10) | 232 (17.11) |
|  | Hyposmia or anosmia | 1975 (86.78) | 155 (51.50) | 90 (29.90) | 56 (18.60) | 301 (13.22) | 1285 (86.07) | | 107 (51.44) | 64 (30.77) | 37 (17.79) | 208 (13.93) | | 1175 (86.65) | 94 (51.93) | 55 (30.39) | 32 (17.68) | 181 (13.35) |
|  | Head and facial pain or pressure | 1966 (86.38) | 158 (50.97) | 96 (30.97) | 56 (18.06) | 310 (13.62) | 1294 (86.67) | | 104 (52.26) | 65 (32.66) | 30 (15.08) | 199 (13.33) | | 1183 (87.24) | 93 (53.76) | 53 (30.64) | 27 (15.61) | 173 (12.76) |

**TABLE E4. Combined disease of the SRAR, PDAR, and PIAR groups**

| **Variable** | **SRAR** | | **PDAR** | | **PIAR** | |
| --- | --- | --- | --- | --- | --- | --- |
|  | **Yes (%)** | **No (%)** | **Yes (%)** | **No (%)** | **Yes (%)** | **No (%)** |
| Asthma(Physician‐diagnosed) | 234 (10.28) | 2042 (89.72) | 189 (12.66) | 1304 (87.34) | 177 (13.05) | 1179 (86.95) |
| Hypertension | 203 (8.98) | 2057 (91.02) | 95 (6.41) | 1388 (93.59) | 72 (5.34) | 1277 (94.66) |
| Arrhythmia | 139 (6.12) | 2132 (93.88) | 59 (3.96) | 1432 (96.04) | 48 (3.55) | 1306 (96.45) |
| Previous myocardial infarction | 26 (1.15) | 2239 (98.85) | 14 (0.94) | 1471 (99.06) | 12 (0.89) | 1336 (99.11) |
| Stable angina pectoris | 24 (1.06) | 2238 (98.94) | 12 (0.81) | 1474 (99.19) | 9 (0.67) | 1340 (99.33) |
| Diabetes | 67 (3.06) | 2119 (96.94) | 32 (2.22) | 1411 (97.78) | 22 (1.68) | 1288 (98.32) |
| Chronic stomach disease | 243 (10.71) | 2025 (89.29) | 128 (8.59) | 1362 (91.41) | 110 (8.13) | 1243 (91.87) |
| Chronic kidney disease | 41 (1.83) | 2200 (98.17) | 24 (1.63) | 1450 (98.37) | 20 (1.49) | 1319 (98.51) |
| Chronic liver disease | 62 (2.81) | 2144 (97.19) | 29 (1.99) | 1426 (98.01) | 24 (1.82) | 1296 (98.18) |
| Hyperlipidemia | 204 (9.01) | 2061 (90.99) | 97 (6.52) | 1390 (93.48) | 78 (5.77) | 1274 (94.23) |
| Cerebrovascular disease | 58 (2.56) | 2208 (97.44) | 22 (1.48) | 1467 (98.52) | 16 (1.18) | 1336 (98.82) |
| Thyroid disease | 189 (8.34) | 2076 (91.66) | 112 (7.53) | 1375 (92.47) | 95 (7.04) | 1255 (92.96) |
| Anxiety and depression | 91 (4.03) | 2166 (95.97) | 40 (2.70) | 1442 (97.30) | 34 (2.53) | 1312 (97.47) |
| Eczema/dermatitis | 560 (25.37) | 1647 (74.63) | 409 (28.21) | 1041 (71.79) | 378 (28.70) | 939 (71.30) |
| Sinusitis | 486 (21.35) | 1790 (78.65) | 322 (21.57) | 1171 (78.43) | 285 (21.02) | 1071 (78.98) |
| Nasal polyp | 88 (3.90) | 2167 (96.10) | 59 (3.99) | 1421 (96.01) | 50 (3.72) | 1295 (96.28) |
| Nasal surgery | 41 (1.83) | 2198 (98.17) | 24 (1.63) | 1451 (98.37) | 18 (1.34) | 1323 (98.66) |
| Chronic bronchitis | 120 (5.36) | 2117 (94.64) | 68 (4.61) | 1407 (95.39) | 59 (4.40) | 1281 (95.60) |
| Self-reported food allergy reaction | 438 (19.30) | 1832 (80.70) | 349 (23.41) | 1142 (76.59) | 338 (24.96) | 1016 (75.04) |
| Self-reported drug allergy reaction | 246 (10.87) | 2018 (89.13) | 152 (10.23) | 1334 (89.77) | 140 (10.36) | 1211 (89.64) |
| History of skin itching | 583 (25.85) | 1672 (74.15) | 403 (27.23) | 1077 (72.77) | 364 (27.10) | 979 (72.90) |

**TABLE E5. Cognitive situation of patients in SRAR, PDAR, and PIAR groups**

| **Variable** | | **SRAR** | | **PDAR** | | **PIAR** | |
| --- | --- | --- | --- | --- | --- | --- | --- |
|  |  | **Number (n=2276)** | **Percentage（%）** | **Number  (n=1493)** | **Percentage（%）** | **Number (n=1356)** | **Percentage（%）** |
| How to understand allergy? | Comprehensive anti allergy prevention and control activities /Internet/ Media | 522 | 22.93 | 352 | 23.58 | 324 | 23.89 |
|  | Hospital doctors | 1338 | 58.79 | 904 | 60.55 | 822 | 60.62 |
|  | Family or friends | 1212 | 53.25 | 808 | 54.12 | 733 | 54.06 |
| What disease was initially thought to be the cause of the illness? | Believe in catching a cold | 1358 | 59.67 | 893 | 67.52 | 816 | 60.18 |
|  | Believe in allergy | 669 | 29.39 | 485 | 32.48 | 443 | 32.67 |
| Does the work or study area have artemisia removal? | No | 2270 | 99.74 | 1491 | 99.80 | 1353 | 99.78 |
|  | Yes | 6 | 0.26 | 3 | 0.20 | 3 | 0.22 |
| Does the residential or living area have artemisia removal? | No | 2272 | 99.82 | 1491 | 99.87 | 1354 | 99.86 |
|  | Yes | 4 | 0.18 | 3 | 0.13 | 2 | 0.14 |

**TABLE E6. Treatment of patients in SRAR, PDAR, and PIAR groups**

| **Variable** | | **SRAR** | | **PDAR** | | **PIAR** | |
| --- | --- | --- | --- | --- | --- | --- | --- |
|  |  | **Number (n=2276)** | **Percentage (%)** | **Number (n=1493)** | **Percentage (%)** | **Number (n=1356)** | **Percentage (%)** |
| Treatment | Antihistamines | 960 | 42.18 | 734 | 49.16 | 690 | 50.88 |
|  | Antibiotic (oral or intravenous) | 225 | 9.89 | 155 | 10.38 | 141 | 10.40 |
|  | Montelukast sodium | 419 | 18.41 | 332 | 22.24 | 317 | 23.38 |
|  | Nasal corticosteroid therapy | 618 | 27.15 | 471 | 31.55 | 444 | 32.74 |
|  | Nasal antihistamine spray | 314 | 13.80 | 237 | 15.87 | 222 | 16.37 |
|  | Nasal decongestant spray | 104 | 4.57 | 72 | 4.82 | 67 | 4.94 |
|  | Anti-allergy eye drops | 512 | 22.5 | 424 | 28.4 | 408 | 30.09 |
|  | Antibiotic eye drops | 275 | 12.08 | 219 | 14.67 | 212 | 15.63 |
|  | Oral corticosteroids | 180 | 7.91 | 142 | 9.51 | 138 | 10.18 |
|  | Folk remedies | 153 | 6.72 | 115 | 7.70 | 109 | 8.04 |
|  | Traditional Chinese Medicine Treatment | 331 | 14.54 | 239 | 16.01 | 218 | 16.08 |
|  | Acupuncture | 78 | 3.43 | 59 | 3.95 | 53 | 3.91 |
|  | Allergen immunotherapy | 18 | 0.79 | 15 | 1.00 | 15 | 1.11 |
|  | Monoclonal antibody | 18 | 0.79 | 12 | 0.80 | 12 | 0.88 |
|  | Nasal irrigation | 432 | 18.98 | 341 | 22.84 | 318 | 23.45 |
|  | Mucus solubilizer | 19 | 0.83 | 15 | 1.00 | 10 | 0.74 |

1. † SRAR, self-reported allergic rhinitis [↑](#footnote-ref-1)
2. ‡ PDAR, physician‐diagnosed allergic rhinitis [↑](#footnote-ref-2)
3. § PIAR, pollen-induced allergic rhinitis [↑](#footnote-ref-3)
